# COVID-19 in Patients with Transfusion Dependent Thalassemia (TDT) in Indonesia: Characteristics of the Disease and Patients, and Comparison between Epidemiological Data for COVID-19 and Thalassemia in Indonesia and Southeast Asia

**DOI:** 10.3390/hematolrep14010002

**Published:** 2022-02-23

**Authors:** Tubagus Djumhana Atmakusuma

**Affiliations:** Division of Hematology-Medical Oncology, Department of Internal Medicine, Faculty of Medicine, Universitas Indonesia, Jakarta 10430, Indonesia; hom_fkui@yahoo.com; Tel.: +622180675479

**Keywords:** COVID-19, blood transfusion, transfusion-dependent thalassemia, HbE, beta-thalassemia

## Abstract

Background: People living with transfusion dependent thalassemia have a high risk of becoming infected with COVID-19. This can be caused by both internal factors, namely the formation of alloantibodies and autoimmune disorder, and external factors such as routine visits for blood transfusions. Chronic complications of thalassemia also render them more vulnerable to infectious diseases, including COVID-19. However, anecdotal data shows that thalassemia patients experience less incidence of COVID-19 compared to the general population. Purpose: This study aims to find the correlation between COVID-19 in thalassemia-dependent transfusion patients in Indonesia and Southeast Asia. Patients and Methods: This study used a cross-sectional design. The study was conducted at the Division of Hematology and Medical Oncology of the Cipto Mangunkusumo Hospital in Jakarta from May 2020–August 2021. The total sampling method was used involving all thalassemia major patients who had been infected with COVID-19 (obtained directly from medical record and through the thalassemia patients-parents foundation). Results: From 10,397 patients with thalassemia, 67 (0.64%) people were infected by COVID-19 and 2 (2.9%) were deceased. Meanwhile, the incidence of COVID-19 in the general population of Indonesia was 0.87% (more than in the thalassemia population). This means that thalassemia might provide additional protection against COVID-19 due to several mechanisms. This phenomenon has also been seen in other countries with a high prevalence of thalassemia, wherein there are less COVID-19 cases despite the pandemic. On the contrary, countries with low rates of thalassemia had experienced deadly surges of the pandemic. Conclusion: Indonesia and other countries with a high prevalence of thalassemia have lower COVID-19 incidence than countries with low prevalence of thalassemia. Thalassemia might provide additional protection against COVID-19. Well-designed studies are needed to provide better evidence on the protective effect of thalassemia on COVID-19.

## 1. Introduction

COVID-19 is a disease caused by coronavirus. It causes various symptoms, ranging from mild to severe, that generally attack the respiratory tract [1]. This disease has become a global pandemic. Even though those with transfusion-dependent thalassemia represent a high-risk group, there have been no reports concerning thalassemia patients who were infected by COVID-19. Patients with thalassemia major generally have a higher risk of viral and bacterial infections. The occurrence of alloantibody and autoimmune disorders s a result of thalassemia might impair the body’s immune system. Patients with transfusion dependent thalassemia (TDT) routinely visit the hospital to receive blood transfusions every two to five weeks. During the COVID-19 pandemic there has been a blood donor shortage at blood banks, which has deterred patients from meeting their transfusion targets. Since they find it difficult to get an adequate blood volume in a single visit, visits to transfusion services has become more frequent (sometimes up to once a week). Routine blood transfusion is also a risk factor for COVID-19 infection due to increased exposure during the trip from home to hospital, during the blood transfusion itself, and when travelling back home from the hospital.

Every day in our clinic, there are approximately 300 adult TDT patients and 600 pediatric patients who receive regular transfusions. There are no nationwide data regarding this in Indonesia. Indonesia is located in the ‘thalassemia belt’, with a high prevalence of thalassemia beta HbE and beta major. There are about 10.397 TDT patients managed in 56 centers [2]. The prevalence of HBe thalassemia has been estimated to be around 1.5–3.6%.

In several countries, such as Italy [3], Cyprus, and several Southeast Asian countries, the number of cases of thalassemia patients affected by COVID-19 is lower than the general population. In our clinic, there were only three patients with transfusion-dependent thalassemia who were infected with SARS-CoV2 in 2020 (despite the incremental increase in national daily cases). Based on the data above, there seems to be a discrepancy between the incidence of COVID-19 in patients with thalassemia compared to the general population. Therefore, we wanted to study the characteristics of thalassemia patients and their correlation with thalassemia and COVID-19 epidemiological data, further investigated the possibilities which can cause a decreased incidence of COVID-19 in thalassemia patients.

## 2. Materials and Methods

Diagnosis of COVID-19 was based on rt-PCR result. Blood group type data was obtained from various blood banks. The clinical severity of COVID-19 was determined according to the severity of symptoms. The type of thalassemia was obtained from the medical records which were derived from the result from Hb analysis. We gave oral and written explanation of the purpose, benefits, and research procedures to the subjects. Appropriate consent was obtained and signed electronically due to contact restriction during COVID-19 pandemic. Data were processed using SPSS 26 for Windows. This study has obtained ethical approval from the Medical Ethics Commission of Faculty Medicine of Universitas Indonesia. This study was conducted in accordance with the declaration of Helsinki.

## 3. Results

There were 10,397 registered thalassemia patients with regular visits for blood transfusions in Indonesia. In our thalassemia center in Jakarta, we found eight patients with COVID-19. Nationwide data were obtained from POPTI, a well-established organization which involves the parents of thalassemia patients. The foundation’s members consist primarily of parents since people with thalassemia are often diagnosed at early childhood.

From March 2020–August 2021, we obtained data from 38 provinces, and 67 (0.64%) thalassemia patients were confirmed with OVID-19. Two patients (2.9%) died. The highest number of thalassemia cases with COVID-19 was from Jakarta. The characteristics of thalassemia patients infected with COVID-19 in Indonesia and the relation between their variables can be seen in Table 1 and Table 2.

**Figure 1:**
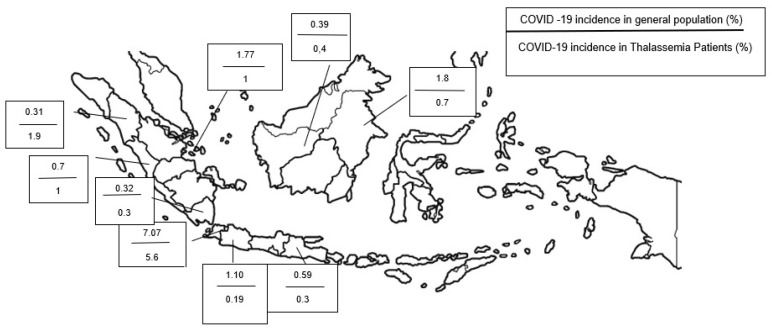
COVID-19 incidence in general population compared to COVID-19 incidence in thalassemia patients.

Figure 1 shows that COVID-19 incidence in thalassemia patients was generally lower than in the general population. In provinces with a high prevalence of thalassemia, such as Aceh (0.012%), the incidence of COVID-19 in the province was only 0.3%. This contrasts with other province with low prevalence of thalassemia, such as East Borneo (0.004%), which has an incidence of COVID-19 six-fold greater than that of Aceh’s (1.8%). Detailed data on the incidence of COVID-19 in each province in Indonesia can be seen in Table 3.

## 4. Discussion

In this nationwide study, we obtained more than ten thousand people living with thalassemia and conducted a survey to determine the impact of COVID-19 pandemic. Due to COVID-19 travel and contact restrictions, data were collected using online methods. We successfully contacted thalassemia representative in 34 provinces and found that during March 2020–August 2021, there were 67 thalassemia patients who were infected by COVID-19. Most of the cases originated from the capital Jakarta which was also the epicentrum of the pandemic. Meanwhile, Indonesia is an archipelago with vast genetic diversity especially in terms of hemoglobinopathies. Therefore, by obtaining detailed data from every province, we could get an overview of how thalassemia might be related to reduced incidence of COVID-19.

Table 1 shows that most of the patients were aged between 3–18 years and 19–40 years old. We divided the subjects into these age range since children under 18 years old are managed by pediatricians, while those above 18 years old are managed by internist/adult hematologists. Our data showed that most of the patients were in this age bracket. This is due to a more active lifestyle and mobility. We only had several subjects older than 40 years old since a lot of patients could not survive beyond 40 years due to the complications of thalassemia.

Table 1 also showed that Betawi, Javanese, and Sundanese represent the ethnicities most infected by COVID 19. These are the most common ethnic groups in Indonesia (especially in Jakarta). Although there are hundreds of ethnicities in Indonesia, those three are the most prevalent (especially in the Java Island). In order to obtain association of ethnicity with COVID-19, more subjects from each ethnicity are needed.

We performed an analytical test between patient’s characteristics and COVID-19 status (survive vs. deceased), as shown in Table 3. We can conclude that there is no relationship between the variables except for clinical severity of COVID-19. The percentage of deceased subject with moderate COVID-19 symptoms (33%) is quite high, which indicates the variable as statistically significant. In the entire group of subjects, a total of two people were deceased. The reported cause of death was due to active TB infection and chronic liver disease and not due to COVID-19 itself.

Our data show that thalassemia patients displayed lower incidence of COVID-19 compared to general population. For example, in the province of East Borneo, the percentage of COVID-19 cases in thalassemia patient was less than fifty percent of the incidence in the general population. Even in Jakarta, which was the most affected province with diverse ethnicity, the rate of COVID-19 in thalassemia was still below the general population.

Moreover, the prevalence of thalassemia, including those undetected, silent carriers, seems to protect the host from COVID-19. In regions where there is high prevalence of thalassemia, it is rational to hypothesize that the number of thalassemia minor or silent carriers is also higher. From Figure 1, we can obtain data regarding the proportion of COVID-19 in general population compared to in thalassemia patients. In most regions of Indonesia, the incidence of COVID-19 in thalassemia patients is generally lower than general population. However, we also found that there are some provinces with higher COVID-19 incidence in thalassemia compared with the general population. This is caused by the low number of registered thalassemia patients compared to other regions, thereby increasing the percentage of thalassemia patients affected by COVID-19 at the specific area due to the smaller denumerator.

Nevertheless, thalassemia is not the only factor associated with the spread of COVID-19. In areas with high population density and high mobility, such as Jakarta, the incidence of COVID-19 is the highest in the country. However, our study showed that even in the “COVID hotspots”, people with thalassemia had lower SARS-CoV-2 infection rate compared to general population.

From these data, it seems probable that thalassemia might provide additional protection against SARS-CoV2 infection. In other words, theoretically a region with a high prevalence of thalassemia should be less affected by COVID-19. Based on data collected from several countries in Southeast Asia (ASEAN Countries), countries with a high prevalence of thalassemia such as Vietnam, Cambodia, and Laos were less affected by COVID-19 rates compared to countries with low prevalence of thalassemia carrier such as Western countries (as can be seen at Table 4).

Table 4 depicts the number of COVID-19 cases and thalassemia prevalence in ASEAN countries. The thalassemia prevalence in the table represents a rough estimate of the latest numbers. We were unable to obtain the number of confirmed COVID-19 cases in other countries since ours is the first publication reporting COVID-19 in thalassemia patients. However, from Table 4, it can be inferred that countries with high prevalence of beta thalassemia have reduced the incidence of COVID-19. These findings are similar to those published by de Sanctis, et al. [7] which shows that the proportion of beta-thalassemia patients being infected with COVID-19 was higher than other types of thalassemia, but that the total number of thalassemia patients infected by COVID-19 is less than the general population. Nevertheless, the study collected data from the UK, Iran, and Italy. Our study was the first in the Asian thalassemia population.

A study conducted by Edouard Lansiaux et al. [6] showed that higher prevalence of beta thalassemia and higher resistance against COVID-19 was found in the areas of Puglia, Sardinia, Sicilia, Italy, Cyprus, Sardinia, and Southeast Asia [6,8]. This geographical pattern, known as the ‘thalassemia belt’, spans along countries from the Mediterranean Ocean to the Asia continent. In Cyprus, according to our data, there were 560 people with beta thalassemia major. Of these 560 patients, 33 (5.8%) people were affected by COVID-19. Among these, the mortality rate was 0%, while the average hospitalization day ranged from 3 to 10 days. All patients recovered without being admitted to ICU or receiving ventilator support [3]. In Italy, there are several studies concerning thalassemia and COVID-19. The incidence of COVID-19 in thalassemia patients was 0.00057%, with a mortality rate is 2% [3]. This numbers were very low compared to the general population in Italy, a country which was severely devastated by COVID-19.

Another study by Papadopoulos KI et al. [8] supported our findings in Table 4. Concerning the spread of COVID-19, in areas with high HbE thalassemia prevalence, such as Thailand, Cambodia, and Laos, the incidence of COVID-19 was lower [9]. In Indonesia, there was an island name Sabu Island which has the highest prevalence of beta HbE thalassemia. This phenomenon has been studied by Weatherall et al. [9] since this island’s inhabitants have markedly high rate of Beta HbE thalassemia. As expected, the incidence of COVID-19 in this region is lower (0.44%) compared to national incidence (1.07%) and much lower to peak incidence in the capital (7.07%) [10]. In these two provinces in Indonesia, the percentage of COVID-19 patients is lower than in other regions.

However, it should be also taken into account that population mobility plays an important role in the spread of COVID-19. People on Sabu Island are significantly more isolated than those living in Jakarta. Therefore, although thalassemia itself might protect them from COVID-19, we should also consider their isolated location as the reason why the incidence of COVID-19 is lower in this population.

Mortality due to COVID-19 in thalassemia patients is 2.9%. Meanwhile the mortality by COVID-19 in Indonesia as of 18 July 2021 is 2.5%. The high death rates in thalassemia patients affected by COVID-19 might be affected by several factors, such as organ dysfunction due to chronic complication of thalassemia, inadequate iron chelation therapy, and the unavailability of specialized COVID-19 referral hospital for such patients.

Theoretically, thalassemia patients are more prone to have moderate to severe clinical severity. However, our data showed that the incidence is lower than general population. The lower incidence of COVID-19 in thalassemia is possibly due to a defect in the beta-globin chain, thereby inhibiting the pathogenetic mechanism of SARS-CoV-2 infection [9]. Moreover, our data showed that most thalassemia patients (84.8%) had asymptomatic or mild infection. An epidemiology study by Lansiaux et al. [5] showed that beta-thalassemia might provide protection against COVID-19. A study conducted by Wenzhong et al. [11] has shown that proteins in COVID-19 such as (open reading frame) ORF1ab, ORF3a, and ORF10 have tendency to attack beta chain hemoglobin to form porphyrin. Porphyrins is needed for viruses to survive. Furthermore, the lungs fail to exchange carbon dioxide and oxygen due to the dysfunction of hemoglobin [12]. In thalassemia patients, the lack, or even absence, of the beta chain prevents the virus from entering host cells, rendering the host less susceptible [5].

Studies on immune competence in beta-thalassemia have revealed numerous quantitative and functional defects involving T and B lymphocytes, immunoglobulin production, neutrophils and macrophages, chemotaxis, and phagocytosis, as well as the complement system [13]. In non-thalassemic patients, in vitro cell experiments show that delayed release of cytokines and chemokines occurs in respiratory epithelial cells, dendritic cells (DCs), and macrophages at the early stage of SARS-CoV infection. Later, the cells secrete low levels of interferons (IFNs) and high levels of proinflammatory cytokines (interleukin (IL)-1 β, IL-6, tumor necrosis factor (TNF), and chemokine [14]. Therefore, in patients with thalassemia, the cytokine storm cascade will be attenuated due to the incompetence on immunity that will affect the expression of cytokines.

Thalassemia patients have higher risk of exposure to SARS-CoV-2 due to the frequent hospital visits for regular blood transfusions [15]. They are also burdened by chronic complications which attenuate their immune system, such as iron overload and splenectomy [15]. Excessive transfusions can lead to the accumulation of iron, ferritin, and hemosiderin in the blood [16]. Iron overload can also occur due to ineffective erythropoiesis and increased intestinal iron absorption. This has been implicated as the main precipitating factor of immune deficiency in thalassemia. Iron directs the immune response toward a T-helper (Th)-2 response pattern, which is unfavorable for fighting bacterial or viral infection. Cellular iron availability also modulates the differentiation and proliferation of Th1 and Th2 cell subsets, partly related to the different dependence of cells on transferrin-mediated iron uptake [17]. Theoretically this attenuated immune response should render patient with thalassemia vulnerable to SARS-CoV-2 infection. However, the data in our study and other studies showed otherwise. Therefore, there might be an alternative mechanism explaining why people with thalassemia are somehow “more immune” to SARS-CoV-2.

As a comparison, countries with low prevalence of thalassemia carriers seem to be more affected by COVID-19. In Brazil, COVID-19 affected 8.9% of the population, with a mortality rate of 0.2%, and a 1.8% prevalence of thalassemia carriers. Another example comes from one of the most affected countries by COVID-19, India, where the number of COVID-19 patients exceeded thirty million people. This country has low prevalence of beta thalassemia carriers. From these two countries, it can be seen that a low number of thalassemia carriers can be associated with a high number of COVID-19 cases [4].

The United Kingdom has experienced several waves of COVID-19, resulting in an explosion in the number of cases. This monarchy has an extremely low number of thalassemia carriers. Moreover, despite nationwide vaccination strategies, the United States has also suffered from multiple waves of the pandemic. According to Weatherall et al. [9], thalassemia patients in the US are scarce. These facts therefore support the theory that low number of thalassemia carriers leads to higher cases of COVID-19.

Looking at the pattern described above, there is one country which showed atypical presentation. The ‘ground zero’ of COVID-19, the People’s Republic of China (PRC), has a low prevalence of thalassemia carriers. However, despite being the first country affected by COVID-19, they can manage the pandemic well. This could be caused by a one-way government system, an extensive tracing system, a massive vaccination system, and a high level of population awareness in preventing the transmission of COVID-19.

In Table 4, the prevalence of alpha thalassemia, beta thalassemia, and HbE thalassemia in Malaysia were higher than that in Indonesia. However, the total number of COVID-19 cases in general population is higher than in Malaysia. This might be due to several factors. First, Indonesia’s population is almost ten times of Malaysia, with its population spread throughout the archipelago. There are some isolated islands where COVID-19 did not cause significant cases (especially in the furthest islands from the capital). Moreover, the prevalence of thalassemia in Indonesia might be underestimated, due to unavailability of Hb electrophoresis and genetic testing throughout the country. There is estimated to be a higher number of undiagnosed thalassemia carriers in Indonesia. In addition, Malaysia has high testing rate for COVID-19 (ranked third in the ASEAN countries). On the other hand, Indonesia ranked ninth due to its huge population and large distribution of people among its islands.

Several previous studies had also investigated the association between blood type and susceptibility to COVID-19 infection. Zietz et al. [18] found that people with blood group A had higher susceptibility to COVID-19 compared with blood group O. In general, people with O type blood are less vulnerable than those with non-O type blood. It is hypothesized that polymorphism in the ABO gene plays an important role in natural immunity against SARS-CoV-2. Our data show a high proportion of non-ABO subjects with COVID-19, far higher than the normal blood group distribution in the general population. This might support the hypothesis that blood types play a role in susceptibility to COVID-19.

From these data above, we can obtain valuable insight regarding the risk of contracting COVID-19 in people living with thalassemia. In general, countries with high prevalence of thalassemia have lower incidence of COVID-19 and lower mortality rate. Other factor that should be considered is the government system along with their respective healthcare policies.

## 5. Conclusions

Indonesia, along with other countries with a high prevalence of thalassemia, has had a lower COVID-19 incidence than western countries with a low prevalence of thalassemia. Our data showed that in the Indonesian population, thalassemia might provide additional protection against COVID-19. Since this phenomenon can be explained by theoretical possibilities, well designed studies are needed to provide better evidence of the protective effect of thalassemia against COVID-19.

## Figures and Tables

**Table 1:** Characteristics Thalassemia patients infected by COVID-19 in Indonesia.

General Characteristic	*N* = 66
Gender, *n* (%)	
Male	29 (43.3)
Female	37 (56.7)
Age, *n* (%)	
3.0–18.0	30 (44.8)
19.0–40.0	26 (38.8)
41.0–60.0	10 (16.4)
Blood Type, *n* (%)	
A (+)	20 (29.9)
AB (+)	10 (14.9)
B (+)	14 (20.9)
O (+)	23 (34.3)
Ethnicity, *n* (%)	
Bataknese	1 (1.4)
Bengkulu	1 (1.4)
Betawi	14 (20.8)
Buginese	1 (1.4)
Chinese	2 (2.9)
Javanese	24 (35.8)
Lampung	1 (1.4)
Medan	2 (2.9)
Malay	2 (2.9)
Minang	1 (1.4)
Sundanese	18 (26.8)
Thalassemia Type, *n* (%)	
Alfa 3 deletion	2 (3.0)
Beta Intermedia	1 (1.5)
Beta HbE TDT	19 (28.4)
Beta Mayor	44 (65.7)
Beta minor	1 (1.5)
COVID-19 clinical severity, *n* (%)	
Asymptomatic	31 (46.3)
Mild	32 (47.8)
Moderate	4 (6.0)
Severe	0 (0.0)
Outcome, *n* (%)	
Deceased	2 (2.9)
Recovered	65 (97.1)
Vaccination Status Prior to COVID-19 infection, *n* (%)	
Already	3 (4.2)
Not Done	66 (91.7)

**Table 2:** Relationship between patient’s characteristics and COVID-19 status.

Patient’s Characteristics	COVID-19 Status		*p*-Value
	Survive	Deceased	
Gender, *n*			
Male	29	0	0.318
Female	36	2	
Age Group, *n*			
3–18	30	0	0.300
19–40	25	1	
41–60	10	1	
Blood Type, *n*			
A (+)	19	1	0.374
AB (+)	14	0	
B (+)	9	1	
O (+)	23	0	
Thalassemia Type, *n*			
Alfa 3 deletion	2	0	0.898
Beta Intermedia	1	0	
Beta HbE TDT	19	0	
Beta Mayor	42	2	
Beta minor	1	0	
COVID-19 Clinical Severity			
Asymptomatic	30	1	0.021
Mild	32	0	
Moderate	3	1	
Severe	0	0	
Vaccination Status Prior to COVID-19 Infection			
Already	3	0	0.912
Not Done	62	2	

**Table 3:** Recapitulation of thalassemia in each province and COVID-19 TDT patients [2,4].

No	Province	Total Population	Total TDT Patient	Prevalence of TDT/Population	Total COVID Cases in General Population	TDT Patients with COVID-19	TDT Patients with COVID-19 (%)	Mortality in TDT Patients with COVID-19
1	ACEH	5,274,871	631	0.0120%	20,949	0	0%	0
2	NORTH SUMATRA	14,799,361	162	0.0011%	46,053	3	1.9%	1
3	WEST SUMATRA	5,534,472	22	0.0004%	61,350	0	0%	0
4	RIAU	6,394,087	97	0.0015%	83,628	0	0%	0
5	JAMBI	3,548,228	54	0.0015%	16,443	0	0%	0
6	SOUTH SUMATRA	8,467,432	332	0.0039%	37,917	0	0%	0
7	BENGKULU	2,010,670	100	0.0050%	14,003	1	1.0%	0
8	LAMPUNG	9,007,848	316	0.0035%	29,078	1	0.3%	0
9	BANGKA BELITUNG ISLANDS	1,455,678	117	0.0080%	26,778	0	0%	0
10	RIAU ISLANDS	2,064,564	97	0.0047%	36,581	1	1.0%	0
11	DKI JAKARTA	10,562,088	864	0.0082%	746,306	49	5.6%	1
12	WEST JAVA	48,274,162	4164	0.0086%	530,806	8	0.19%	0
13	CENTRAL JAVA	36,516,035	1449	0.0040%	329,216	0	0%	0
14	DI YOGYAKARTA	3,668,719	165	0.0045%	92,085	0	0%	0
15	EAST JAVA	40,665,696	695	0.0017%	239,168	2	0.3%	0
16	BANTEN	11,904,562	654	0.0055%	92,746	0	0%	0
17	BALI	4,317,404	23	0.0005%	61,175	0	0%	0
18	WEST NUSA TENGGARA	5,320,092	26	0.0005%	16,331	0	0%	0
19	EAST NUSA TENGGARA	5,325,566	0	0%	26,363	0	0%	0
20	WEST KALIMANTAN	5,414,390	234	0.0043%	21,149	1	0.4%	0
21	CENTRAL BORNEO	2,669,969	38	0.0014%	30,347	0	0%	0
22	SOUTH BORNEO	4,073,584	157	0.0039%	40,029	0	0%	0
23	EAST KALIMANTAN	3,766,039	137	0.0036%	96,564	1	0.72%	0
24	NORTH KALIMANTAN	701,814	0	0%	16,286	0	0%	0
25	NORTH SULAWESI	2,621,923	0	0%	19,718	0	0%	0
26	CENTRAL SULAWESI	2,985,734	0	0%	16,792	0	0%	0
27	SOUTH SULAWESI	9,073,509	61	0.0007%	72,240	0	0%	0
28	SOUTHEAST SULAWESI	2,624,875	0	0%	13,817	0	0%	0
29	GORONTALO	1,171,681	0	0%	6948	0	0%	0
30	WEST SULAWESI	1,419,229	0	0%	7113	0	0%	0
31	MALUKU	1,848,923	0	0%	12,127	0	0%	0
32	NORTH MALUKU	1,282,937	0	0%	8204	0	0%	0
33	WEST PAPUA	1,134,068	0	0%	15,904	0	0%	0
34	PAPUA	4,303,707	0	0%	23,826	0	0%	0
	Total	270,203,917	10,397	0.089%	2,908,040	67	11.21%	2

**Table 4:** Prevalence of COVID 19 and thalassemia in ASEAN countries [5,6].

No	Country	Total Population	Number of COVID-19 Cases	COVID-19 Cases/1 Million Population (*n*)	Alpha Thalassemia (%)	Beta Thalassemia (%)	HbE Thalassemia (%)	TDT Patient Mortality due to COVID-19
1	Indonesia	270,203,917	2,908,040	10,762	0.5%	3%	1–25%	7
2	Philippines	104,900,000	1,605,762	15,308	5.4%	1.2%	1%	N/A
3	Vietnamese	95,540,000	165,339	1731	0.05%	1.6–25%	1–73%	N/A
4	Thailand	69,400,000	652,185	9397	5.5–30%	1–9%	5–50%	N/A
5	Myanmar	53,370,000	306,354	5740	10.5%	4%	1–26%	N/A
6	Malaysia	31,200,000	1,163,291	37,285	1.8–7.5%	3–5%	5–46%	N/A
7	Cambodia	16,010,000	79,051	4938	10%	3%	31–63%	N/A
8	Laos	7,364,903	7015	952	42%	9%	24–48%	N/A
9	Singapore	5,612,000	65,213	11,620	2.91%	0.93%	0.64%	N/A
10	Timor Leste	1,339,862	10,982	8196	N/A	N/A	N/A	N/A
11	Brunei	428,607	338	789	4.3%	0.02%	0.01%	N/A
	Total	655,369,289	6,963,570	106,718				

Notes: N/A, not available.

## Data Availability

Additional data can be requested from corresponding author if needed.
